# Principles of dormancy evident in high-grade serous ovarian cancer

**DOI:** 10.1186/s13008-022-00079-y

**Published:** 2022-03-23

**Authors:** Trevor G. Shepherd, Frederick A. Dick

**Affiliations:** 1 London Regional Cancer Program, London Health Sciences Centre, London, ON N6A 5W9 Canada; 2 Department of Obstetrics & Gynaecology, Western University, London, ON N6A 5C1 Canada; 3 Department of Pathology and Laboratory Medicine, Western University, London, ON N6A 5C1 Canada; 4 Children’s Health Research Institute, London, ON N6A 4V2 Canada

**Keywords:** Tumor dormancy, High-grade serous ovarian cancer, Spheroid, Metastasis, Cellular quiescence, Stress adaptation signaling, Autophagy, Tumor microenvironment, Minimal residual disease

## Abstract

In cancer, dormancy refers to a clinical state in which microscopic residual disease becomes non-proliferative and is largely refractory to chemotherapy. Dormancy was first described in breast cancer where disease can remain undetected for decades, ultimately leading to relapse and clinical presentation of the original malignancy. A long latency period can be explained by withdrawal from cell proliferation (cellular dormancy), or a balance between proliferation and cell death that retains low levels of residual disease (tumor mass dormancy). Research into cellular dormancy has revealed features that define this state. They include arrest of cell proliferation, altered cellular metabolism, and unique cell dependencies and interactions with the microenvironment. These characteristics can be shared by dormant cells derived from disparate primary disease sites, suggesting common features exist between them.

High-grade serous ovarian cancer (HGSOC) disseminates to locations throughout the abdominal cavity by means of cellular aggregates called spheroids. These growth-arrested and therapy-resistant cells are a strong contributor to disease relapse. In this review, we discuss the similarities and differences between ovarian cancer cells in spheroids and dormant properties reported for other cancer disease sites. This reveals that elements of dormancy, such as cell cycle control mechanisms and changes to metabolism, may be similar across most forms of cellular dormancy. However, HGSOC-specific aspects of spheroid biology, including the extracellular matrix organization and microenvironment, are obligatorily disease site specific. Collectively, our critical review of current literature highlights places where HGSOC cell dormancy may offer a more tractable experimental approach to understand broad principles of cellular dormancy in cancer.

## Introduction

Epithelial ovarian cancer is the 7th most common cancer in women worldwide. It is difficult to treat because it is most often diagnosed at an advanced stage with metastases present [1]. Collectively, epithelial ovarian cancer includes less aggressive histotypes such as low-grade serous, endometrioid, clear cell, and mucinous (type 1), and more aggressive high-grade serous ovarian cancer (type 2) [2]. High-grade serous ovarian cancer (HGSOC) makes up more than 70% of cases, and combined with its more rapid spread, it disproportionately contributes to morbidity and mortality of ovarian cancer [3]. HGSOC is typically treated with surgical debulking and adjuvant chemotherapy consisting of carboplatin and paclitaxel [3]. In addition, targeted agents such as bevacizumab and PARP inhibitors have improved outcomes [1, 3, 4]. While often initially responsive to chemotherapy, emergence of resistance from minimal residual disease (MRD) has emphasized the need to understand the biology and survival strategies of relatively rare HGSOC cells following treatment [3, 5]. Therefore, this review will focus primarily on HGSOC disease characteristics and how they resemble or contrast with cancer dormancy in other disease sites.

**Fig. 1:**
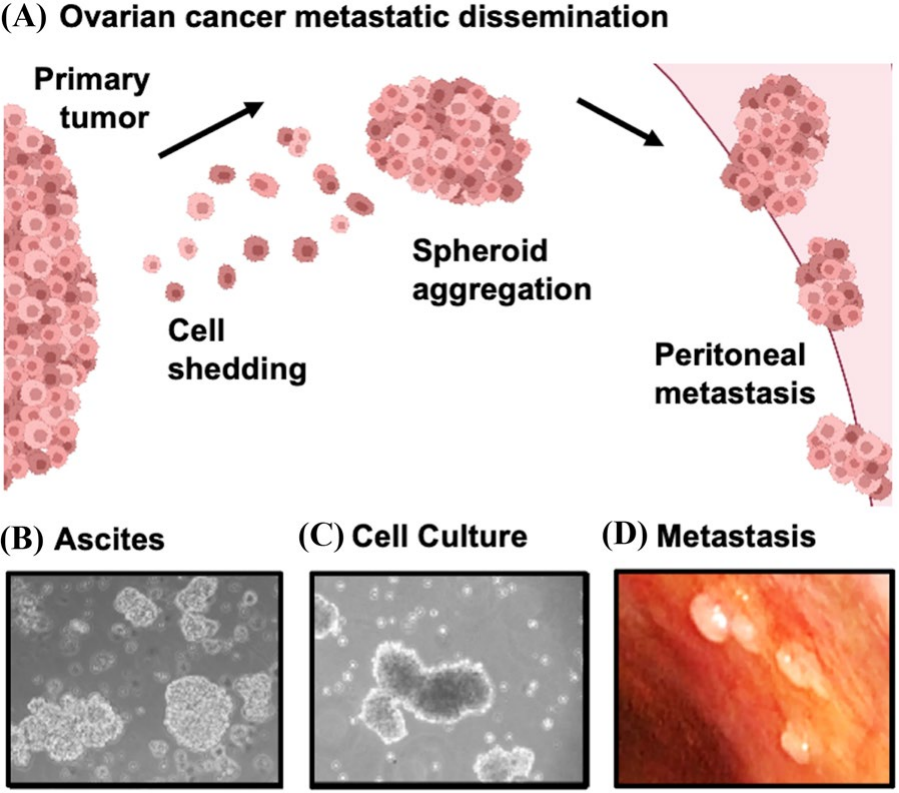
Intraperitoneal dissemination of high-grade serous ovarian cancer. **A** Malignant cells are shed from the primary tumor into the peritoneal space. This often occurs within the accumulated ascites fluid in advanced stage HGSOC. The ability of metastatic HGSOC cells to exist as multicellular clusters, called spheroids, affords malignant cells with enhanced survival characteristics. The induction of a dormancy phenotype thereby protects them from chemotherapeutic insult. Spheroids have an enhanced capacity to reattach to the mesothelial surfaces of the peritoneal cavity to seed secondary tumor deposits and re-initiate cell growth and invasion. **B**,** C** Phase contrast microscopic images of spheroids filtered from HGSOC patient-derived ascites fluid (**B**) and spheroids generated by in vitro suspension culture (**C**). **D** Image of secondary tumor deposits visible on the peritoneal wall of an HGSOC patient undergoing laparoscopic surgery. Photo in (**D**) courtesy of Dr. Dominique Lanvin

The metastatic spread of HGSOC is distinct from most forms of human cancer in that cells are shed from the fallopian tubes and ovaries into the peritoneal space where they aggregate and disseminate to other organs both locally in the pelvic region and beyond to destinations higher in the abdominal cavity (Fig. 1) [1, 5]. Isolation of cancer cell aggregates from the ascites of ovarian cancer patients reveals a non-proliferative population of cells with presumptive dormancy characteristics [6], suggesting they are a likely source of spread and therapeutic resistance. Cell culture models of HGSOC spheroids have revealed mechanisms of reduced proliferation, altered cellular metabolism with distinct survival and dissemination strategies [7–10]. These further suggest that HGSOC harbors a form of dormancy that is relevant to the clinical course of this disease.

Definitions of cancer cell dormancy vary in the literature with some defining it as minimally as the inhibition of proliferation in cancer cells [11]. In a broader sense, dormancy encompasses a host of cellular characteristics that include new metabolic strategies, survival mechanisms, stem-like properties, and distinct microenvironment contacts [12, 13]. Notably, distinct interactions with dormancy supporting microenvironments are key to longevity of cells in the context of breast or prostate cancer cells in bone [14, 15]. In this review, we consider current evidence for dormancy in HGSOC and how it compares with other paradigms of dormancy. We focus on the concept of cellular dormancy, as opposed to tumor mass dormancy [12, 13], as it best matches the biology of spheroids in HGSOC. Cancer cell dormancy in which pre-malignant cells disseminate from an early lesion and disseminate to distant tissues before the primary tumor forms is an important paradigm in breast cancer [16]. This scenario is less consistent with the spread characteristics of HGSOC as most patients are diagnosed with late-stage disease where the primary tumor and metastatic spread are simultaneously present [5]. Interestingly, rare HGSOC patients are first diagnosed with disseminated disease in which a primary tumor is not detectable, clinically referred to as primary peritoneal carcinoma [1]. This suggests that premalignant cells may disseminate very early leading to the appearance of advanced-stage HGSOC without a primary tumor [17, 18]. While a thought-provoking parallel to breast cancer, further discussion is outside the scope of this review that aims to analyze cellular dormancy characteristics of the most common course of HGSOC disease progression.

Perhaps the biggest challenge in dormancy research is the scarcity of the cells being studied [17, 18]. This has resulted in arduous progress to understand these cells under the best of circumstances in breast and prostate cancer where commonly colonized distant tissues such as bone, harbour rare cells [14]. Although some data indicates metastasis may be more efficient when cellular aggregates disseminate [19], most paradigms of metastasis and dormancy are based on solitary cells. HGSOC has distinct features from breast and prostate cancer disease progression that make their identification in clinical samples less of a challenge. Furthermore, modelling growth-arrested, and presumptively dormant, HGSOC cells in culture is more tractable because of in vitro models of spheroids and metastasis [7, 20]. For this reason, HGSOC may offer opportunities to advance our understanding of dormancy principles that are applicable to other disease sites and this motivates our critical review of dormancy data as it pertains to HGSOC in comparison with other cancer types.

The motivation to understand the basic biology of spheroids in HGSOC is to develop better treatment approaches to eliminate this key source of resistance. As detailed in other paradigms of dormancy, the prospect of ‘awakening’ dormant cells to re-sensitize them to chemotherapies that are designed for proliferating cells possesses concerning drawbacks that have been reviewed elsewhere [14, 21]. Alternatively, therapeutic strategies to reinforce dormancy exist, but still eventually lead to relapse [22]. For this reason, we rationalize research into dormancy as seeking to decipher survival dependencies whose removal kills these cells without the resumption of disease progression. The following sections explore distinct dormancy characteristics that support survival and growth arrest. These categories are organized and discussed with the goal of finding new adjuvant therapeutic opportunities and to highlight promising areas for further investigation.

## Quiescence, or slowly proliferating cells, in dormancy

Two hallmarks of cancer described by Hanahan and Weinberg relate to cell cycle control [23]. Self-sufficiency for growth signals and the evasion of negative growth cues describe two conceptual inputs into the cell cycle that are altered in most cancers. Cellular dormancy in which quiescence, or greatly slowed proliferation, are attained in cells derived from advanced-stage disease implies that some means of negative growth control is retained or can be acquired. Furthermore, dominant growth-promoting signals can be overridden by these negative signalling pathways as they are capable of blocking proliferation to support entry into cellular dormancy. This counter-intuitive growth arrest is likely triggered by stress imposed on cancer cells during dissemination [13, 14], or in the case of HGSOC, during release from the primary tumor into peritoneal space where spheroids form.

**Fig. 2:**
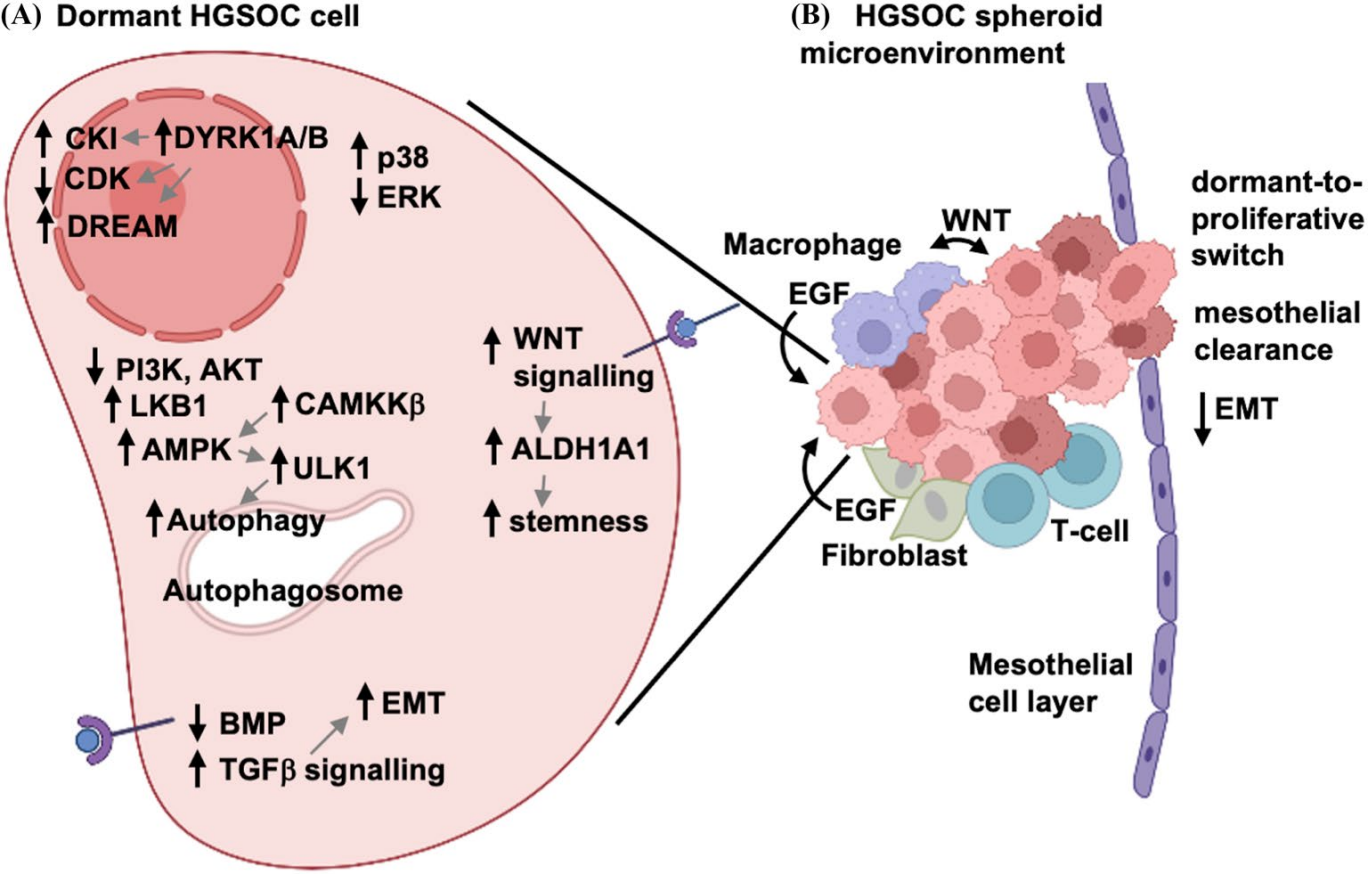
Mechanisms controlling cellular dormancy in HGSOC spheroids. **A** HGSOC spheroid cells undergo several stress induced reprogramming events to induce cellular quiescence through p38 and ERK regulation. These lead to increased expression of CDK inhibitors, assembly of DREAM, and inhibition of CDK activity. Additional pathways contribute to the dormancy phenotype, including metabolic reprogramming via increased LKB1 and AMPK activities, decreased PI3K-AKT signaling, and the induction of macroautophagy. EMT and stemness are promoted by TGFβ and WNT signaling, respectively, and these ligands can be produced directly by HGSOC cells or by other cells within the microenvironmental niche. **B** HGSOC spheroids may be directly impacted by numerous different cell types of the unique microenvironment of the peritoneal cavity and malignant ascites fluid. These include tumor-associated macrophages, fibroblasts, and T-cells, all of which can provide cytokine signals or direct cell-cell contacts to impact the dormant phenotype. Spheroids directly interact with mesothelial cells on peritoneal surfaces, leading to mesothelial clearance and invasion into the underlying stroma. During this process, HGSOC cells can undergo a dormant-to-proliferative switch, as well as reverse their mesenchymal phenotype when establishing secondary tumor deposits

A basic principle of dormant cancer cell signaling in proliferative control that mirrors the hallmarks reasoning above is that pro-growth signals through the Ras-MAPK pathway are often downregulated as evidenced by lowered phospho-ERK. Simultaneously, the stress responsive MAPK, p38 is activated and has been shown to induce growth arrest mechanisms. This core signaling change has been reported in dormancy examples derived from multiple solid tumor types [24–26], and this signaling switch has been demonstrated in cell culture models of HGSOC as well (Fig. 2A) [27].

Reduced cell proliferation implies that dormancy cues block cyclin/CDK activity, reduce Myc levels, and induce other features of quiescence. Experimental cell culture models of dormancy reveal increased levels of CDK inhibitor proteins specifically in growth-arrested conditions [14, 28, 29]. In addition, in vivo xenograft models have long used histological staining to demonstrate increased levels of CDK inhibitor proteins p21, p27, and p16 both as a marker of dormant cells and to characterize their growth-inhibited status [30]. This data is derived from cancer cells representing breast, melanoma, and squamous carcinoma cancer types. In some cases, transcriptional upregulation of CDK inhibitor genes suggests dormancy-triggered gene expression patterns control this effect [14, 29, 31]. Other studies demonstrate the role of F-box protein regulation as controlling dormancy through stabilized CDK inhibitor expression [32], suggesting post-translational mechanisms also can impinge on CDK regulation in dormancy.

Similar experimental evidence for CDK inhibition of proliferation exists for HGSOC-derived cells and spheroids (Fig. 2A). Reduced proliferation and spheroid formation is accompanied by elevated levels of p27 and coincident reduced phospho-SKP2 [7], suggesting a post-translational mechanism induces CDK inhibition. Extension of this cell culture model of dormancy combined with siRNA knock down has revealed the role of numerous CDK inhibitors in HGSOC dormancy [33]. CDKN1 family members appear to be required most often across a panel of HGSOC cell lines, but some low passage patient-derived cultures also showed dependencies for CDKN2 family members [33].

Consistent with stress-dependent signals contributing to dormancy, the survival kinases DYRK1A/B are implicated in dormant cell cycle arrest and maintenance of viability [14]. DYRK kinases are often regulated by protein expression and possess active states that are independent of T-loop phosphorylation [34], suggesting expression levels may be the best indicators of involvement in dormancy. DYRK1 kinases are capable of inhibiting cell proliferation through a number of targets including Cyclin D degradation [35, 36], p27 stabilization [37], and DREAM [dimerization partner (DP), retinoblastoma (RB)-like, E2F and MuvB] complex assembly through phosphorylation of the LIN52 protein [38]. DYRK1A expression is relatively widespread, while specific cases of ovarian and pancreatic cancer possess amplification of the *DYRK1B* gene and dependence on it rather than *DYRK1A* [33, 37, 39]. Assembly of DREAM is coincident with increased expression of the RB family protein p130, a marker of quiescence [33, 38]. Collectively, these studies suggest an important role for DYRK1 kinase family members in a stress-responsive induction of quiescence. Intriguingly, this role for DYRK kinases in cancer dormancy has been developed largely through the study of ovarian cancer cells and a parallel role in solitary dormant cells from breast or prostate cancers has yet to be reported.

The *MYC* oncogene is overexpressed or hyperactivated in numerous cancer types [40], including HGSOC [41, 42]. Its activity is incompatible with quiescence and its expression is largely absent in dormant cancer cells, or in residual disease that is therapy resistant. Downregulation of *MYC* in a number of experimental models induces dormancy and chemotherapy resistance [43–46]. MYC regulatory pathways that use FBXW7 or NFATC4 to lower its expression in dormancy are also emerging in the literature [32, 47]. Given MYC’s critical role in proliferation and necessity for inactivation in quiescence, there remains much to be learned about its regulation in dormancy.

The widespread importance of negative growth control in dormancy suggests it is an appealing target for inactivation to eradicate residual disease. However, its removal suggests that oncogenic pathways that fuel proliferation may restore cell cycle progression, leading to malignancy. In vitro studies of DYRK1A and DREAM component loss in ovarian cancer cell dormancy indicate that a brief episode of DNA replication is followed by rapid cell death with no evidence of sustained proliferation [33]. Furthermore, loss of DREAM assembly in a conditional adult mouse mutant indicates loss of its contribution to cellular quiescence in normal tissues is tolerated without evidence of proliferation [48]. These studies suggest that targeting negative growth control may be feasible as an anti-dormancy therapeutic approach. Ultimately, specific agents need to be tested in reliable pre-clinical models of dormancy and this type of work remains unreported.

## Signaling pathway dependencies in dormancy

The PI3K-AKT-mTOR pathway is one of the most widely mutated and activated signaling pathways in human cancers. It has the capacity to promote cell proliferation, mRNA translation, block apoptosis, enhance cell motility and migration, enhance epithelial-mesenchymal transition, and modulate autophagy, to name just a few [49]. However, entry into dormancy is commonly accompanied by reduction in this signaling pathway. In breast cancer dormancy, disseminated tumor cells were found to have low Ser473 phospho-AKT, even when isolated for culture and re-expanded [50]. Similarly, dormant squamous carcinoma cells have reduced AKT phosphorylation while utilizing a different pathway to activate mTOR, thus allowing proliferative signaling from PI3K to be dampened for cellular dormancy, yet maintaining cell viability [51]. Studies on colon cancer cells suggest that entry into dormancy as opposed to undergoing apoptosis are highly dependent on reduction but not complete loss of AKT signaling [52]. Overall, PI3K effects on AKT are down-regulated in dormancy while survival signals from mTOR are often sustained.

Mutations in the PI3K-AKT-mTOR pathway, such as activating missense mutations in *PIK3CA* or *PTEN* deletions, are observed infrequently in HGSOC [42]. However, other copy number events do give rise to heightened PI3K signaling capacity in the majority of HGSOC tumors [42]. PI3K signaling activities mediated by AKT are consistently and robustly decreased in spheroids [7]. Although AKT activity could be expected to support HGSOC cell survival while in suspension to block anoikis, its counter-intuitive downregulation is essential to drive at least two key phenomena in HGSOC spheroids: cellular quiescence and autophagy [7, 53, 54].

There have been, and continue to be, many clinical trials using PI3K/mTOR inhibitors, including those for epithelial ovarian cancers. This strategy is likely driven by the preponderance of activating driver mutations in this pathway among non-HGSOC ovarian histotypes [2]. However, the experimental preclinical data in HGSOC spheroids argues this therapeutic strategy may induce tumour dormancy [7]. Since these inhibitor strategies are often tested in patients in the recurrent platinum-resistant setting, the appropriate window-of-opportunity may have already been missed. It is also possible that PI3K/mTOR pathway inhibition could prolong progression-free survival by supporting dormancy; however, prolonged maintenance therapy using such targeted agents could yield the emergence of additional mechanisms of resistant disease beyond that of platinum resistance already prevalent in ovarian cancer [55].

As malignant cells detach from a primary tumour to spread to distant sites, they commonly trigger stress responses. The inherent cellular mechanisms implicated in HGSOC metastasis may be different from other cancers due to its unique progression within the peritoneal cavity. Metastatic HGSOC cells trigger intracellular stress signaling when they detach into suspension, as well as being deprived of growth factors, ECM components, nutrients, and oxygen. A key pathway that monitors these stressors is the Liver Kinase B1 (LKB1) and AMP-activated protein kinase (AMPK) signaling pathway (Fig. 2A). AMPK acts as a crucial hub kinase that reprograms the overall metabolism of cancer cells by shifting away from anabolic metabolism to a more catabolic phenotype [56]. Thus, AMPK signaling yields new substrates for energy production in cancer cells that are energy- and substrate-depleted. AMPK activation typically requires low ATP levels to facilitate the allosteric shift in its catalytic subunit T-loop and subsequent threonine phosphorylation. Indeed, HGSOC spheroids possess reduced intracellular mitochondrial redox potential and ATP levels, and a concomitant increase in AMPK phosphorylation and activity [27, 57]. However, HGSOC spheroid cells do not utilize canonical LKB1 activity to phosphorylate AMPK, but rather calcium-calmodulin dependent kinase kinase beta (CAMKKβ) [27], even though LKB1 is essential for spheroid survival [27, 57]. A major downstream effect mediated by AMPK activity in EOC spheroids is the induction of macroautophagy. AMPK expression and activity, particularly that controlled by CAMKKβ, are required for induction of autophagy in HGSOC spheroids [58]. AMPK activity also acts to control cell proliferation by inducing cytostasis under nutrient-limiting conditions [57]. Cytostasis by AMPK activation occurs in HGSOC cells within spheroids and it likely contributes to the dormancy phenotype in addition to the observed AKT downregulation.

Although the stress signaling mediator LKB1 may not be required to control AMPK in HGSOC spheroids, it is still crucial for both spheroid cell viability and metastatic progression [27], highlighting an important area for future investigation. At present, the contributions of AMPK and LKB1 to dormancy in other cancer types or sites of dissemination is not known. It is tempting to speculate that mechanistic insights from ovarian cancer dormancy may yield new commonalities of cell signaling events in tumor dormancy more broadly.

## Autophagy and dormancy specific metabolism

Primary tumors across most disease sites utilize glycolysis to fuel growth and proliferation, a phenomenon known as the Warburg effect [23]. Disseminated cancer cells are known to shift from glycolytic metabolism to one dependent on fatty acid oxidation and ultimately oxidative-phosphorylation for energy needs [59, 60]. Not surprisingly, these cells are also metabolically dependent on anti-oxidant programs to survive the resulting production of reactive oxygen species [61, 62]. Whether these metabolic adaptations in disseminated cancer reflect true dormancy is unclear. Most literature describing metabolic changes in dormant cancer cells are based on studies of ovarian cancer [14, 22]. They describe a metabolic shift to fatty acid oxidation in HGSOC spheroids and it promotes the dormancy phenotype and increases cell survival in suspension [63]. In a broader tumor dormancy context, it is possible that metabolic changes are a response to the loss of previous nutrient sources in the primary tumor, or the need for alternative nutrients in the new dormant environment [11], raising the question whether different dormancy paradigms are likely to have the same metabolic needs.

Macroautophagy, otherwise termed autophagy, is a general intracellular degradation process of organelles, macromolecules and in some cases pathogens. Typically, autophagy is a universal stress-induced phenomenon under nutrient-depleted and starvation-like conditions to facilitate the generation of alternative substrates for energy production [64]. Autophagy has tumour-suppressive activity in early malignant states as it can drive senescence or cell death. However, it is widely regarded as a key mechanism that can promote cancer cell survival under hypoxia in avascular tumors, and nutrient and growth factor depletion in rapidly-growing tumours, or in the face of chemotherapy and radiotherapy [64].

Autophagy is rapidly induced in HGSOC spheroids (Fig. 2A), and this is driven by two anti-parallel signaling pathways that were introduced in the signaling section above, namely the downregulation of AKT and the upregulation of AMPK [53, 58]. More recently, a primary target of AMPK, the unc51-like protein kinase 1 (ULK1) within the autophagy-initiation complex (AIC), was shown to be activated and required for survival in HGSOC spheroids [65]. Autophagy is necessary to sustain cell survival within spheroids, but it has wider implications since it is known to promote chemotherapy resistance under dormancy-like conditions in other cancers. In addition, autophagy has been directly implicated in ovarian tumor growth during metastasis, particularly through the expression and activity of the tumor suppressor *ARHI*, a RAS family protein whose signaling can maintain a dormancy-like state of xenografted tumor cells [66, 67].

When considered together with the section on signaling mechanisms above, a number of important gaps in our knowledge are evident. First, it is unclear if, or to what extent, autophagic mechanisms are controlled through PI3K-AKT and AMPK-ULK1 in cancer dormancy paradigms other than ovarian. Similarly, dependence on fatty acid metabolism and neutralization of reactive oxygen species is suggested by various aspects of HGSOC spheroid biology, but it is not established as it is in other paradigms of residual disease. For example, it is reported that lipid desaturation is critical to the biology of stem-like spheroid cells in HGSOC, which may be implicated in tumor dormancy [68]. Future efforts in this area offer promise to reveal a more unified view of common metabolic principles in cellular dormancy.

Given the challenges of PI3K pathway inhibition described above, autophagy potentially offers a more direct consequence of cell signaling in dormancy as a therapeutic target. The majority of clinical trials targeting autophagy in advanced human cancers use hydroxychloroquine, likely because of its known safety profile from use in malaria prophylaxis and rheumatoid arthritis [69]. Hydroxychloroquine is often tested in combination with chemotherapy or radiation since these are standard therapies that have been shown to induce cytoprotective autophagy. Unfortunately, there are limitations to using general lysosomal-targeting agents in this context, and few clinical trials have shown any beneficial effects of autophagy inhibition through hydroxychloroquine use [70]. Given the function of autophagy in maintaining cell survival under dormancy-like conditions in MRD, perhaps future pre-clinical studies should address the importance of direct autophagy inhibition specifically in contexts of dormancy rather than progressive disease.

## Epithelial-to-mesenchymal transition and stem cell characteristics in dormancy

The transforming growth factor-beta (TGFβ) superfamily of cytokines has widespread implications in human cancers, serving contextual tumour suppressive and pro-metastatic roles [11]. In general, TGFβ family members (including bone morphogenetic proteins) have been shown to be produced in bone niches where their signals suppress proliferation and stimulate dormancy [71–73]. In the context of disseminated cancer cells these signals can be interpreted as EMT supporting [11], thus allowing these cells an adaptable phenotype that can facilitate metastasis.

Studies using HGSOC spheroids have observed the reciprocal expression and activity between bone morphogenic protein (BMP) and TGFβ signalling (Fig. 2A), with the former being decreased in spheroids and the latter being increased [74, 75]. These reciprocal signaling activities are required for efficient spheroid formation and integrity, likely through the induction and maintenance of epithelial-mesenchymal plasticity [75]. Akin to the dormant-to-proliferative switch mediated by differential AKT activity in spheroids [7], TGFβ signalling control of this EMT phenotype is reversible upon spheroid reattachment [75]. This capacity of reattaching spheroids may explain how metastatic HGSOC cells possess epithelial marker expression in both primary and secondary tumours, but more mesenchymal markers in spheroids during active dissemination [6]. We propose that this plasticity is crucial for efficient spread and establishment of secondary lesions in the unique peritoneal environment of advanced HGSOC.

Rare dormant cancer cells that are capable of initiating new clinically-detectable metastases suggest dormancy and stem cell-like phenotypes go hand-in-hand. Recent work on disseminated cancer cells revealed that negative regulation of WNT signaling suppresses proliferation and prevents immune detection in perivascular niches [76]. This discovery suggests that regulatory pathways relevant to stem cells contribute to the behavior of disseminated cancer cells. In ovarian cancer, there is emerging evidence of stem-like properties in at least some cells present in HGSOC spheroids. Desaturated fatty acid accumulation has been shown to be associated with higher expression of SOX2, Nanog, and OCT4 stem cell transcription factors and the marker ALDH1A1 in ovarian cancer spheroids [68]. Inhibition of fatty acid desaturase enzymes diminishes NFκB activity and expression of these stem cell markers. Expression of ALDH1A1 is similarly dependent on β-catenin expression specifically in spheroids and not in adherent monolayer culture [77, 78]. Inhibition of ALDH1A1 enzymatic activity with experimental small-molecule inhibitors kills spheroid cells [77, 79], further suggesting dormant spheroids contain cancer stem-like cells, which are crucial to promote metastasis.

A theme throughout this review is that common pathways exist to control dormancy that are similar between HGSOC and other disease sites. TGFβ family and WNT signaling pathways contribute to key elements of EMT and stem cell biology in dormancy. Again, there are distinct aspects of how these pathways function in HGSOC spheroids that raise the question of applicability of findings in one disease site paradigm to another. However, tumor dormancy studies across disparate disease sites suggest that TGFβ and WNT signaling pathways are candidates for therapeutic intervention that target dormancy. Thus, appreciating the subtle mechanistic differences in disease site specific aspects of tumor dormancy are likely critical to the successful use of dormancy-directed therapeutics in the future.

## Microenvironment and immune cell interactions with dormant cells

Research into dormant cancer cell niches highlight three main locations for these cells to reside [22]. The perivascular niche is a common location whose proximity to capillary beds suggests that disseminated cancer cells reach this location following extravasation from the bloodstream. Within bones, circulating cancer cells can compete with hematopoietic stem cells and occupy their niche [22]. Data indicates that they can compete for the same ligand-receptor interactions, as CXCR4 blockade disrupts interactions with CXCL12 and releases both dormant breast cancer cells and hematopoietic stem cells into circulation [80–82]. Lastly, also within bones, dormant cells derived from solid or hematogenous primary tumors can occupy the osteoblast niche [15]. In addition to physical interactions with the niche, dormant cancer cells interact with the immune system to escape detection and survive in these locations [22].

It is difficult to see commonalities among these examples as each microenvironment involves different cell-cell contacts and signaling events mediated by distinct cytokines [22]. The complexity and specificity of these dormant cell-niche specific interactions is perhaps best illustrated by the opposing roles of closely-related TGFβ family members between different niches. Specifically, perivascular niches established by resident endothelial cells in lung utilize Thrombospondin-1 to induce quiescence in newly-disseminated breast cancer cells [83, 84]. Eventually vascular sprouting activates TGFβ1, that in turn stimulates the resumption of proliferation by these dormant cancer cells [84]. Alternatively, head and neck squamous cell carcinomas disseminated to bone rely on TGFβ2 for entry into quiescence and disruption of this signaling axis induces metastatic outgrowth in this location [71].

Dormant cancer cells in all locations share a common outcome of escaping eradication by the immune system [14]. In some cases, interactions with immune cells serve to shape the dormant microenvironment. Growth-arrested cancer cells in a number of disease site scenarios are known to down-regulate expression of class I MHC [22]. This conceals cancer cells from the actions of cytotoxic immune cells that may normally be activated by the ‘foreign’ epitopes cancer cells display. In addition, T-regulatory cells and macrophages can contribute to an immune-suppressed microenvironment through interferon gamma (IFNγ) production [14, 22], and IFN signaling can contribute to growth suppression in cancer cell dormancy [14]. Given the complexity of specific dormant microenvironments occupied by rare disseminated cells [15], it is not surprising that the dormant niche of HGSOC spheroids is expected to be similarly distinct.

Spheroids from HGSOC patients represent a distinct microenvironment that is mobile and possesses self-contained features essential for survival and dissemination of ovarian cancer (Fig. 2B). Spheroids contain HGSOC cancer cells that are released from the primary tumor site. These are joined by fibroblasts, T-cells, and macrophages that contribute to this dormant cell niche [85, 86]. Several lines of evidence suggest that HGSOC cells and their spheroid-associated macrophages develop a symbiotic relationship that fuels cancer cell survival and ultimately disease progression (Fig. 2A). Macrophages provide WNT and EGF ligands to signal to nearby cancer cells [9, 10], while WNT production by ovarian cancer cells activates M2 macrophages [87]. This contributes to cell-cell adhesion and cancer stem cell phenotypes that aid in spheroid formation and disease dissemination, as well as chemotherapy resistance [88]. Furthermore, immune-suppressive macrophages are recruited to spheroids in a WNT pathway-dependent manner [89]. High levels of WNT ligands in ascites are also predictive of disease progression in ovarian cancer [90]. These studies illustrate an interdependence between macrophages and HGSOC cells. In addition, fibroblasts contribute EGF to HGSOC cells in spheroids further contributing to disease spread [86]. T-cells are also detected in the HGSOC cell microenvironment and their putative attraction by macrophages correlates with longer survival for patients [85]. Furthermore, it has been reported that low CD47 expression on HGSOC stem cells renders them vulnerable to immune clearance, but the bulk of non-stem like HGSOC cells in the spheroid help protect these cells from the resident immune system [91]. Overall, the spheroid microenvironment is a complex structure in which the 3D architecture and distinct cell populations contribute to its biological characteristics in dormancy and dissemination.

A further distinction between HGSOC dormancy and that of other solid tumors is that native spheroid pathobiology enables metastatic dissemination through distinct interactions with mesothelial surfaces in the abdominal cavity. These interactions involve both cell-cell signaling in which HGSOC cells activate the mesothelium to aid in attachment and infiltration [10] and cell-matrix interactions upon mesothelial clearance [5, 92] (Fig. 2B). The generation of myosin-derived forces driven by attaching spheroids assists in clearing mesothelial cells to allow infiltration into the underlying stroma [8]. The omentum is the most common site of HGSOC metastasis, and this organ has many resident cell types, growth factors and nutrients that may impact HGSOC dormancy [5]. Omentum-specific growth factors, such as adipokines, act to reprogram HGSOC cell metabolism via AMPK activity and may be implicated in reversing dormancy-like phenotype to re-establish tumor growth at this secondary site [93, 94]. These examples further illustrate unique aspects of the HGSOC spheroid microenvironment that contribute to the ultimate emergence from dormancy and their resumption of proliferation to create metastases.

## Conclusions and therapeutic implications of targeting dormancy in ovarian cancer

The most significant factor affecting both progression-free survival and overall survival rates among women with advanced HGSOC is achieving the lowest level of residual disease following aggressive cytoreductive surgery and combination chemotherapy [1, 4]. It is certainly possible that additional therapeutic strategies to target and eradicate HGSOC dormant cells within this context of MRD could further improve survival. The key question from this standpoint is how to exploit dormancy as a therapeutic vulnerability to kill residual HGSOC cells or reinforce their dormant phenotype.

Epidemiological data suggests the diabetic drug metformin has a protective role for ovarian cancer [95]; since metformin affects the mitochondrial respiratory chain and stimulates AMPK activity, its mechanism of action may include maintenance of tumor cell dormancy. The complexity of PI3K/mTOR pathway activities may make its general inhibition less useful to promote dormancy in HGSOC, however specific downstream components may be more attractive targets. For example, AKT inhibitors in combination with agents that block autophagy would be more focused on the most relevant aspects of PI3K signaling in dormancy [53]. As new strategies to target dormancy progress to testing, their implementation will require new insights into the resistance mechanisms that may emerge in order for their potential benefits to be judged.

Most research implicates tumor dormancy in the context of advanced stages of disease and as a mechanism to evade therapeutic insult or immune-mediated clearance. However, HGSOC tumor cells have the capacity to disseminate from fallopian tubes early in disease progression, long before dormancy mechanisms can impact treatment resistance. The concept of early dissemination in HGSOC is particularly relevant since evidence exists for cells comprising pre-malignant serous tubal intraepithelial carcinoma (STIC) lesions to spread to the ovary, or in rare cases directly into the peritoneum [18]. A critical question in the etiology of HGSOC is whether these early, fallopian-derived, disseminating cells follow a similar dormancy pathway as in the late-stage dormancy paradigm reviewed here. If they do, then dormancy may represent another critical target when detection of early-stage disease becomes possible. Unfortunately, experimental models of HGSOC initiation that involve murine oviductal epithelium remain relatively rare in the literature [96–99], and access to pre-malignant clinical STIC specimens will also be required to advance research in this area.

The concept of tumor dormancy has wide implications among many human cancers and can impact the ability to attain a durable response to treatment. However, we suggest that reductionist attempts describing it with a single definition, or with a specific set of criteria across all disease sites is counter-productive. In this review, we highlight that there are many common elements of dormancy shared among cancer types, but the underlying molecular mechanisms can often be distinct among different disease sites. Being aware of similarities among dormancy disease site paradigms will likely accelerate our understanding through cross-fertilization of ideas between researchers. At the same time, we conclude from this review that differences among dormancy paradigms are extensive, thus embracing this diversity of discoveries among distinct tumor types is also critical to further advancements in the field. We expect that some knowledge gained through summarizing tumor dormancy in the context of advanced HGSOC will be valuable to investigators studying analogous paradigms in other malignancies and metastatic sites.

## Data Availability

Not applicable.

## Abbreviations

3D: Three-dimensional
ALDH1A1: Aldehyde dehydrogenase 1A1
AMPK: AMP-activated protein kinase
ARHI: Aplasia Ras homolog member I
CAMKKβ: Calcium/calmodulin-dependent protein kinase kinase beta
CD47: Cluster of differentiation 47
CDK: Cyclin-dependent kinase
CDKN: Cyclin-dependent kinase inhibitor
CXCL12: Chemokine (C-X-C) ligand 12
CXCR4: C-X-C chemokine receptor 4
DREAM: Dimerization partner (DP): retinoblastoma (RB)-like, E2F and MuvB
DYRK: Dual-specificity tyrosine-regulated kinase
ECM: Extracellular matrix
EGF: Epidermal growth factor
EMT: Epithelial-to-mesenchymal transition
ERK: Extracellular signal-regulated kinase
HGSOC: High-grade serous ovarian cancer
IFNγ: Interferon gamma
LKB1: Liver kinase B1
MAPK: Mitogen-activated protein kinase
MHC: Major histocompatibility complex
MRD: Minimal residual disease
mTOR: Mammalian/mechanistic target of rapamycin
NFκB: Nuclear factor kappa B
PI3K: Phosphoinositide 3-kinase
siRNA: Small-interfering RNA
SKP2: S-phase kinase-associated protein 2
STIC: Serous tubal intraepithelial carcinoma
TGFβ: Transforming growth factor beta
ULK1: Unc51-like protein kinase 1
WNT: Wingless-related integration site
